# Washability and Electrical Performance Evaluation of Jacquard Conductive Knitted Fabrics Based on Fuzzy Comprehensive Assessment

**DOI:** 10.3390/polym18080934

**Published:** 2026-04-10

**Authors:** Su Liu, Wei Wang, Hui Yang, Jun Wu

**Affiliations:** 1 School of Future Design, Shanghai Institute of Visual Arts, Shanghai 201620, China; 2 School of Textile and Clothing, Shanghai University of Engineering Science, Shanghai 201620, China

**Keywords:** Jacquard conductive knitted fabrics, comprehensive fuzzy logic method, washing, electrical performance, comprehensive evaluation

## Abstract

This study presents a systematic evaluation of 2-layer conductive Jacquard knitted fabrics with a birdseye backing designed for wearable electronic applications. Three sets of samples with 9 different proportions of conductive yarn (27 samples) are designed on a computerized flat-knitting machine, and three indicators (conductive yarn usage ratio, resistance change ratio after washing, and temperature variation) are examined. The 2-layer Jacquard structure enables conductive yarns to form loops on both the technical face and back, thus producing continuous and interlocked conductive pathways. The experimental results show that the proportions of pattern dots for the conductive yarns determine the amount of conductive yarn used in a 2-layer Jacquard structure with the same technical parameters. For the samples with 10–90% pattern dots, the conductive yarn consumption ratio ranges from 34.80% to 65.18%. After 10 washes, resistance change ratio ranges from 27.66~55.54%, which show a moderate electrical stability. After 10 washes, the heating temperature increases by 15.6 to 19.67 °C, which show good thermal properties. Finally, a fuzzy logic evaluation is conducted with objective indicator weights. The findings provide quantitative evidence for the material–structure integration of conductive knitted textiles and support their potential for applications in next-to-skin smart garments.

## 1. Introduction

Conductive knitted fabrics, which are knitted fabrics embedded with conductive fiber/yarn, have been used as sensors [1], supercapacitors [2], and wearable heaters [3], and for communication devices [4], thus enabling wearable health monitoring devices that are durable and comfortable to wear [5], provide protection [6], support independent living [7], and can be used for physiotherapy rehabilitation [8]. With the rapid development of medical, innovative materials and wearable electronics, conductive knitted fabrics have shown considerable potential due to their flexibility [9], real-time monitoring capability [10], and user convenience [11].

As one of the most important methods of manufacturing flexible conductive fabrics, conductive knitting has been the subject of a number of studies. The studies mainly focus on the conductive materials, knitting configuration and function of the conductive knitted fabrics. Rotzler et al. [12] provided a comprehensive review of the use of conductive materials in knitted structures, with particular emphasis on their potential for wearable and smart textile technologies. In terms of structural configuration, most conductive knitted fabrics reported in the current literature are based on single jersey [13], rib [14], and interlock structures [11]. Meanwhile, we have investigated the electrical properties of both simple and complex knitted constructions. Studies have also shown that the structural configuration significantly affects the electrical behavior of conductive fabrics, primarily due to the different conductive paths formed within the different knitted structures [15,16,17]. In addition, the pattern design of Jacquard knitted fabrics can markedly influence their electrical performance, as different Jacquard patterns produce different conductive paths [18,19]. In our previous study, we established a design principle for conductive Jacquard knitted fabrics. We found that when the proportion of two colors is both close to 50%, the result is an exceptional electrothermal performance [20]. In terms of the function of conductive knitted fabrics, first, the development of knitted strain sensors has drawn substantial research interest, which have led to a number of research work in this area that use a wide variety of different techniques to construct strain-sensitive knitted structures. For instance, Zhou et al. [21] developed polyaniline-coated cotton knitted fabric for strain sensing. Abbas et al. [22] developed knitted reinforced structures for health monitoring applications. Conductive fabrics have also been widely used as supercapacitors and wearable heaters, and for electromagnetic shielding functions [23]. For instance, Khadem [24] provided a comprehensive overview of recent developments in functional fabric-based wearable supercapacitors, and highlighted the rapid progress of integrating energy-storage capability directly into textile structures. Maurya et al. [25] systematically investigated how knitting engineering parameters and environmental conditions influence the electrothermal performance of heating textiles intended for therapeutic applications.

Since conductive knitted fabrics have been extensively investigated and recognized for their broad application potential, their wearability and electrical performance have become crucial considerations for both their commercialization and industrial applications. For this reason, some of the previous studies have examined the washing and electrical performances of conductive knitted fabrics. Ghouri et al. [26] conducted a critical review on the wash fastness properties of conductive polymer-coated textiles for wearable electronics. Park et al. [27] investigated the changes in the appearance, color, chemical composition, wettability, and electrical performance of conductive fabrics under the influence of perspiration and washing. Their findings further showed that compared with silver-coated fabrics, conductive knitted fabrics made from silver-coated yarns exhibit superior durability in terms of conductivity, while also maintaining more stable colors, tactile properties, and overall electrical performance. However, most of these research works have focused on coated textiles and not on the fabrics knitted with conductive yarns. The latter exhibit exceptional durability because the conductive materials are embedded within the internal structure of the fabric [28]. Research has also focused on the washing and wearable performances of conductive knitted fabrics. For example, Lam et al. [29] examined the washability and abrasion resistance of illuminative knitted e-textiles that incorporate polymer optical fibers (POFs) and silver-coated conductive yarns. They found that half-cardigan and full-cardigan knitted structures continue to provide an exceptional illumination performance after washing and abrasion, owing to their electrical resistance and openness of the stitching. Repon et al. [30] investigated the stretchability and washability of conductive knitted fabrics. Their results indicated that half-Milano knitted structures exhibit a superior performance, with longer durability of the heating characteristics. However, to date, no research has examined the electrothermal performance of conductive Jacquard knitted fabrics. Yet Jacquard knitting is an important technique that offers enhanced esthetic qualities and improved wearability, so that this fabric is highly relevant for the development of functional conductive textiles [31]. Likewise, existing evaluations of washing and wearing properties are rather limited as they mainly present experimental data for individual aspects without providing a comprehensive or integrated assessment [32].

Therefore, this study conducts a full evaluation of the washability and electrical performances of conductive Jacquard knitted fabrics. Three key evaluation indicators are considered in this study, namely loop consumption ratio of conductive yarn ($x_{1}$), electrical stability after washing ($x_{2}$), and electrothermal behavior ($x_{3}$). The parameter $x_{1}$ represents the proportion of conductive yarn used in the Jacquard structure, which reflects the material consumption of conductive yarn in different pattern designs. The parameter $x_{2}$ describes the resistance variation after repeated washing cycles, indicating the electrical stability of the conductive pathways. The parameter $x_{3}$ corresponds to the steady-state heating temperature under a portable charger, representing the heating performance of the fabric. These indicators are used as the input variables in the fuzzy comprehensive evaluation model. In addition, micro-structural images are recorded to observe the morphological changes of the fabric surface before and after washing; however, these images were used only for qualitative analysis and were not included as quantitative indicators in the fuzzy evaluation model. After that, a fuzzy matrix for membership is established. Fuzzy modeling is a common analysis method in evaluating the performance of textiles fabrics [33]. For example, Kabbari et al. [34] employed fuzzy logic modeling to evaluate and predict the water-and oil-repellency as well as the air-permeability performance of plush knitted fabrics. However, no studies to date have applied fuzzy modeling to evaluate the washing and wearing properties of conductive knitted fabrics. The present study addresses this research gap by proposing a systematic fuzzy-based evaluation framework conductive knitted fabrics, particularly those with Jacquard structures. The proposed method enables the integrated assessment of multiple performance indicators and provides practical guidance for structural and pattern design in conductive textile applications, especially in wearable electronics and smart textile systems.

## 2. Experimental Materials and Methods

### 2.1. Structural and Pattern Design Methods

**Figure 1:**
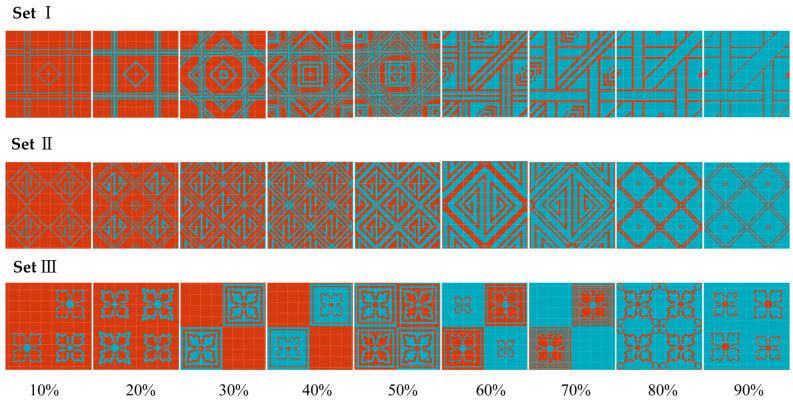
Three different sets of patterns according the percentage of blue dots in a single pattern.

To create a good appearance for the wearable textile, we started with a double jersey Jacquard structure in this study. The details of the structural configurations can be found in our previous study [20]. Based on this structure, a variety of patterns were developed. To ensure that the samples are consistent, all knitted swatches were designed by using the same Jacquard pattern which consisted of 80 wales by 80 courses. Therefore, the patterns were designed by using 80 pattern dots in the horizontal direction and 80 pattern dots in the vertical direction. To comprehensively evaluate the wear ability and electrical performance of the conductive Jacquard knitted fabrics with different amounts of conductive yarns, three different sets were designed according the proportion of blue pattern dots in a single pattern, as shown in Figure 1. The pattern represents the front-side design of the 2-layer conductive Jacquard knitted fabrics, in which the blue dots are knitted with conductive yarns and red dots are knitted with normal yarns in the front layer. From left to right, the proportion of blue dots is 10%, 20%, 30%, 40%, 50%, 60%, 70%, 80% and 90%, respectively. However, it should be noted that the sample labels (10–90%) refer to the designed pattern coverage on the fabric face, rather than the actual percentage of conductive yarn in the fabric. Due to the structural characteristics of double-layer Jacquard knitted fabrics, the conductive yarn not only forms loops on the fabric face according to the Jacquard pattern, but also participates in loop formation on the fabric back following an alternating knitting arrangement [35]. As a result, the actual conductive yarn consumption does not vary linearly with the designed pattern proportion, and the calculated values range approximately from 35% to 65%, as shown in Table 1.

As shown in Table 1, the parameters $L_{c}$ and $L_{n}$ were calculated using STOLL CREATE LITE software version 2.8 (KARL MAYER STOLL Textilmaschinenfabrik GmbH, Reutlingen, Germany), which estimates yarn consumption based on the programmed knitting structure and machine parameters. These values were used for the comparative analysis of conductive yarn consumption among different Jacquard structures. Although these values are theoretical estimations, minor deviations from actual yarn consumption may occur due to knitting conditions. However, such deviations are expected to be consistent across all samples and therefore do not affect the comparative evaluation in this study.

### 2.2. Yarn Selection and Fabric Preparation

Two types of yarns were used in this study, including ordinary yarns and conductive yarns. The specifications of the yarn materials are listed in Table 2. Two ordinary yarns with different colors were used to knit the conductive Jacquard knitted fabrics. In addition, two specifications of silver-plated conductive yarns were employed to construct the conductive patterns in the fabrics. The two conductive yarns were selected based on preliminary experimental screening, which indicated that conductive Jacquard knitted fabrics produced by combining these yarns exhibited favorable heating performance and electrical stability. Therefore, they were chosen for further investigation in this study.

**Figure 2:**
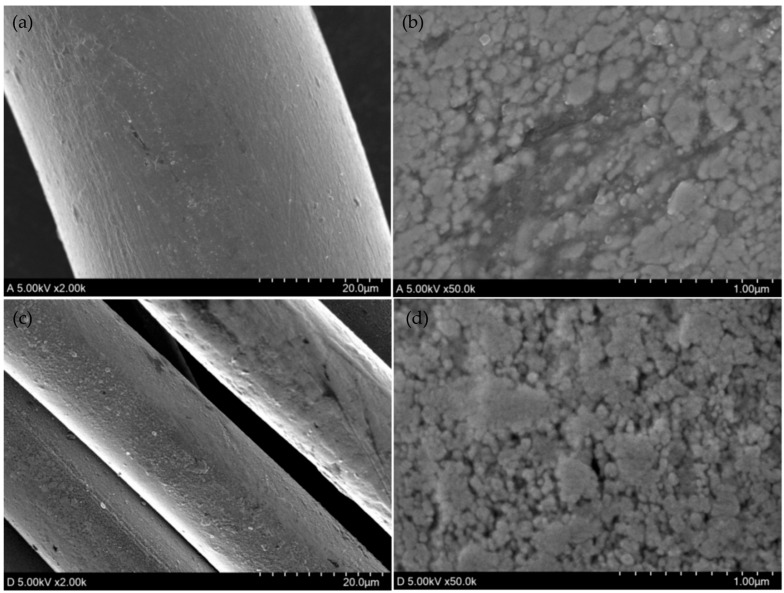
FE-SEM micrographs of (**a**) Conductive Yarn A magnified 2000 times and (**b**) 50,000 times, and (**c**) Conductive Yarn B magnified 1000 times and (**d**) 50,000 times.

Figure 2 presents the FE-SEM micrographs of the two conductive yarns at magnifications of 2000× and 50,000×. Conductive Yarn B (7.78 tex), consisting of silver-coated nylon 66, was used together with ordinary yarns to form the resistive regions of the conductive Jacquard knitted fabrics. Conductive Yarn A (47 tex), also composed of silver-coated nylon 66, was used to knit the electrode regions on both sides.

The combination of ordinary yarns and conductive yarns enabled the fabrication of double-layer conductive Jacquard knitted fabrics with integrated conductive structures.

**Figure 3:**
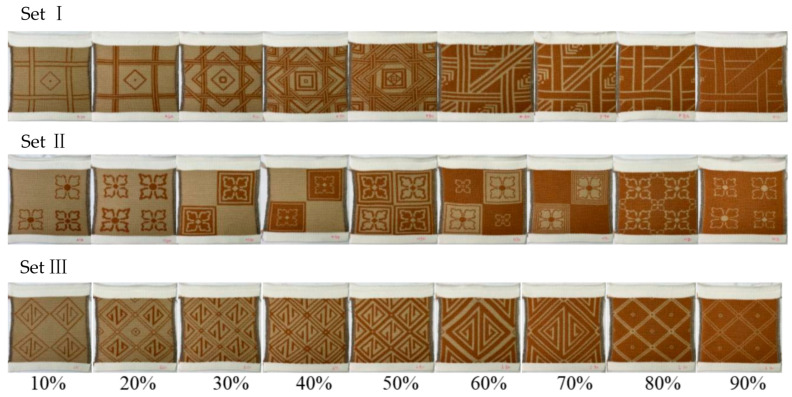
Samples: Sets I, II, and III.

Three sets of conductive Jacquard knitted samples were produced with a 12 gauge computerized flat-knitting machine (Shima Seiki). First, the knitting programs were created by using the SDS-One APEX3 design system (Shima Seiki Mfg., Ltd., Wakayama, Japan) for each individual swatch. The digital patterns were then transferred to the knitting machine for knitting. Prior to the knitting, all of the yarns were conditioned under a standard atmospheric conditions (20 ± 2 °C and 65 ± 5% relative humidity) for 24 h to ensure dimensional stability. Each sample was knitted with the same machine settings, including yarn tension, take-down rate, and knitting speed, to minimize process-induced variability (Table 3). For each sample, three specimens were knitted to ensure experimental accuracy. After knitting, the fabrics were laid flat to relax them for 12 h, followed by dry-steaming, which is a standard finishing process to eliminate residual stress and allow the Jacquard structure to reach equilibrium (Figure 3). All of the fabrics were subjected to the same tests as the aforementioned three samples, and the average values were recorded for the different tests.

### 2.3. Experimental Methods

#### 2.3.1. Wash Testing

**Figure 4:**
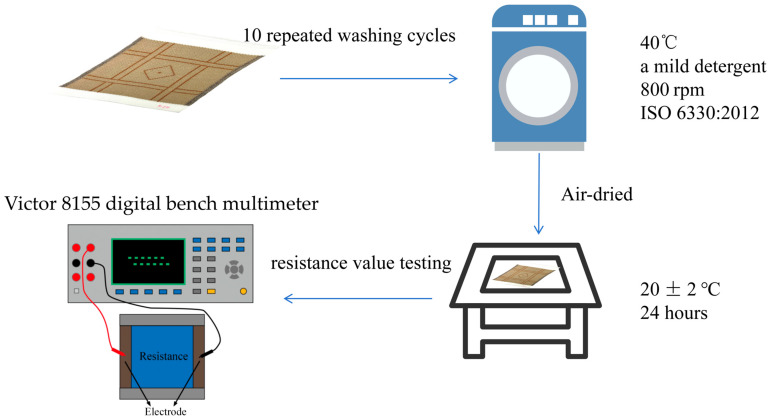
Steps of wash test [36].

After all of the samples were prepared, their washing durability was evaluated. Each sample was subjected to 10 wash cycles. Figure 4 shows the steps of the wash test. The washing was conducted by using a standard laboratory washing machine under controlled conditions, at a constant water temperature of 40 °C, with a mild detergent, and a spinning speed of 800 rpm, in accordance with the standard [36]. After each wash cycle, the samples were air-dried at room temperature (20 ± 2 °C) and conditioned for 24 h before testing. Changes in electrical resistance were measured after each wash cycle to evaluate the durability of the knitted structures. Electrical resistance was measured along the course direction, which corresponds to the current path of the conductive fabric. Prior to electrical resistance measurements, all samples were conditioned under standard laboratory conditions (20 ± 2 °C and 60 ± 5% relative humidity) to minimize the influence of environmental factors. Resistance values were measured using a Victor 8155 digital bench multimeter (Xi’an, China) [20]. All tests were conducted in triplicate to ensure repeatability of the results, and the average values were recorded. In practical applications, wearable heating textiles are typically worn as outer or functional layers and therefore are not subjected to frequent laundering. The wash test data were subsequently analyzed to evaluate the influence of repeated laundering on the electrical properties of the samples.

#### 2.3.2. Electrothermal Performance Evaluation

**Figure 5:**
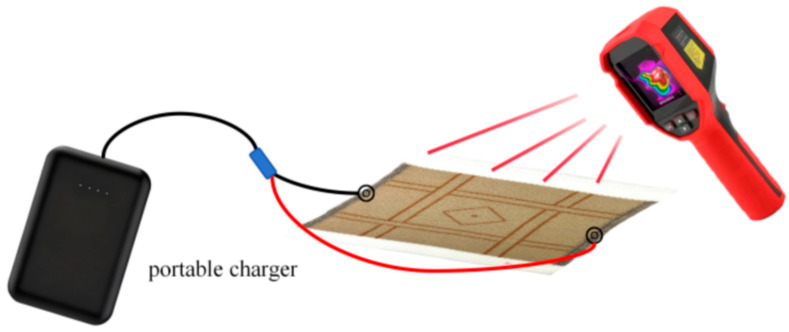
Setup for temperature measurements of samples.

The electrothermal behavior of the fabric was evaluated by applying a voltage and recording the corresponding surface temperature by using an infrared thermal camera as shown in Figure 5. To simulate realistic wearing conditions, the samples were powered using a portable charger (5 V, 3 A, 20,000 mAh power bank), which mimics actual user scenarios where a wearable heating device would be powered by a portable power source. The high-capacity battery ensures stable and long-lasting power output, which enables many hours of continuous operation for wearable heating applications. The 5 V/3 A output supports rapid and consistent delivery of current, which is critical for maintaining a uniform temperature distribution across the conductive fabric. The power bank is compact and lightweight, thus allowing easy integration with wearable prototypes. In addition, built-in safety features, including overcharge, over-discharge, overcurrent, and short-circuit protection, ensure reliable and safe operation during repeated stretching and wash tests. This setup simulates real-life usage scenarios of smart clothing and facilitates an accurate evaluation of the electrothermal performance under practical conditions. The temperature measurements of the samples were carried out by using an infrared thermal imager as shown in Figure 5. The instruments for measuring the electrical properties of the samples are listed in Table 2.

### 2.4. Evaluation Methodology

#### 2.4.1. Conductive Yarn Usage Ratio

To quantify the amount of conductive yarn that can be incorporated into the structure, the ratio ($K_{l}$) for conductive yarn consumption is calculated as follows:

(1) $$K_{l}=\frac{L_{c}}{L_{c}+L_{n}}\times 100\%$$

where $L_{c}$ represents the total conductive loop length in the samples, and $L_{n}$ represents the total normal loop length used in the samples.

#### 2.4.2. Washability Evaluation

The resistance value was measured by using the Victor 8155 digital bench multimeter following ASTM D4496 [37]. For each sample, three repeated measurements were recorded along the course direction. The samples were washed for 1–10 cycles according to ISO 6330:2012 using a mild detergent as discussed in Section 2.4.1. Resistance was measured after each cycle to evaluate conductivity retention. The ratio of the relative change in resistance after ten washes $R_{w}$ is calculated by using:

(2) $$R_{w}=\frac{R_{n}-R_{0}}{R_{0}}\times 100\%$$

where $R_{0}$ is the initial resistance value and $R_{n}$ is the resistance value after n wash cycles.

#### 2.4.3. Thermal Performance Assessment

The heating performance was evaluated by using a portable charger (5 V 3 A 20,000 mAh power bank) as discussed in Section 2.3.2. The surface temperature distribution was obtained via an infrared thermal imaging camera. The temperature variation $T_{v}$ is calculated by using:

(3) $$T_{v}=T_{s}-T_{0}$$

where $T_{0}$ is the initial temperature (20 °C) and $T_{s}$ is the steady-state temperature.

All measurements were carried out under stable laboratory conditions at an ambient temperature of 20 ± 2 °C. The value $T_{0}$ = 20 °C was used as the reference initial temperature in the calculations.

#### 2.4.4. Comprehensive Fuzzy Evaluation Method

##### Procedure of Method

This analysis evaluates the performance of the conductive knitted fabrics by using the entropy weight-comprehensive fuzzy logic method. The evaluation is based on three key indicators ($x_{1}$: conductive yarn usage ratio, $x_{2}$: resistance change ratio after ten washes, $x_{3}$: temperature variation) to grade 27 samples into different categories: excellent, good, average, pass, and fail.

The analysis entailed a three-step methodological procedure. First, the objective weights for each evaluation indicator were determined by using the entropy weighing method. Subsequently, a fuzzy matrix for evaluation was constructed to quantify the degree of membership of each sample across the predefined performance grades. Finally, a comprehensive performance score was derived through the weighted synthesis of these elements, thus enabling a scientific ranking and classification of the samples.

##### Data Normalization

To eliminate dimensional inconsistencies among the indicators, the original data were normalized into the interval [0, 1].

For negative indicators ($x_{1}$ and $x_{2}$), reverse normalization was calculated by using:

(4) $$Z_{ij}=\frac{X_{max}-X_{ij}}{X_{max}-X_{min}}$$

For the positive indicator ($x_{3}$), standard normalization was calculated with:

(5) $$Z_{ij}=\frac{X_{ij}-X_{min}}{X_{max}-X_{min}}$$

where $X_{ij}$ denotes the original value of the j-th indicator for the i-th sample, and $X_{max}$ and $X_{min}$ represent the minimum and maximum values of each indicator, respectively.

##### Determination of Indicator Weights with Entropy Weighing

The entropy weighing method was adopted to objectively determine the weight of the indicators, thus avoiding subjective bias. First, the proportion matrix $P_{ij}$ was calculated based on the normalized data by using:

(6) $$P_{ji}=\frac{X_{ji}}{\sum_{i=1}^{n}X_{ji}}$$

where n is the number of samples, i denotes the sample index, and j represents the indicator index.

Subsequently, the entropy value $e_{j}$ of each indicator was obtained to characterize its degree of dispersion by using Equation (7). Lower entropy values indicate higher discriminative ability.

(7) $$e_{j}=-\frac{1}{\ln n}\sum_{i=1}^{n}P_{ji}\ln\left(P_{ji}\right)$$

When $P_{\mathrm{ji}}$ = 0, the term $P_{ji}\ln\left(P_{ji}\right)$ = 0.

The difference coefficient $g_{j}$ was calculated by using:

(8) $$g_{j}=1-e_{j}$$

Finally, the weight of each indicator $W_{j}$ was determined with:

(9) $$W_{j}=\frac{g_{j}}{\sum_{j=1}^{n}g_{j}}$$

where n is the total number of indicators involved in the comprehensive fuzzy logic assessment.

##### Establishment of Fuzzy Matrix for Evaluation

To construct the fuzzy evaluation matrix, the membership degree of each evaluation indicator with respect to the predefined evaluation grades was first determined using appropriate membership functions. In this study, triangular membership functions were adopted according to the characteristics of the evaluation indicators.

The triangular membership function is defined by using Equation (10).

(10) $$u(x)=\{\begin{array}{c} 0,\;x<a\;or\;x>c\\ \frac{x-a}{b-a},\;a\leq x\leq b\\ \frac{c-x}{c-b},\;b\leq x\leq c \end{array}$$

where *x* represents the value of the evaluation indicator, and a, b, and c are the left, middle, and right endpoints of this level, respectively

Based on these functions, the membership degree $u_{ij}$ was calculated to represent the degree to which the i-th sample belongs to the j-th evaluation grade:

(11) $$u_{ij}=u_{j}(x_{i})$$

where $x_{i}$ is the value of the evaluation indicator for the i-th sample and $u_{ij}$ represents the membership function corresponding to the j-th evaluation grade.

The fuzzy evaluation matrix R was then established by arranging the membership degrees of each sample under the five evaluation grades. In this study, each sample was evaluated across five evaluation grades, resulting in a fuzzy evaluation matrix with dimensions of 3 × 5. The matrix can be expressed as:

(12) $$R=[\begin{array}{ccccc} u_{11} & u_{12} & u_{13} & u_{14} & u_{15}\\ u_{21} & u_{22} & u_{23} & u_{24} & u_{25}\\ u_{31} & u_{32} & u_{33} & u_{34} & u_{35} \end{array}]$$

where each row represents the membership vector of a sample with respect to the five evaluation grades.

In addition, a Kruskal–Wallis non-parametric test was conducted to assess the differences among the three independent sample groups. This method was chosen because of the relatively small sample size and the potential non-normal distribution of the data. A significance level of *p* < 0.05 was employed.

## 3. Results

The fabric densities were measured in accordance with standard textile testing procedures. Specifically, a magnifying pick glass counter was used to observe a defined area of the fabric surface. The number of wales within 10 cm along the horizontal direction was counted, while the number of courses within 10 cm along the vertical direction was recorded. The values for each centimeter were then calculated by dividing the counts over 10 cm by 10. Three measurements were taken at different positions of each sample, and the average values were reported to ensure accuracy and avoid misrepresentations as shown in Table 4. Table 4 shows that with the same knitting parameters, the range of the fabric density of the samples is small (course ranges from 7.8 to 8.3; wale ranges from 10.6 to 11.4).

By incorporating the data on the loop length into Equation (1), the ratio for the usage of conductive yarn ($K_{l}$) is calculated as shown in Table 5. The table shows that $K_{l}$ is very close among the samples with the same proportion of blue dots. This indicates that when the proportion of blue dots remains the same, the consumption of conductive yarn is nearly constant regardless of the pattern. However, the proportion of blue dots has a high impact on conductive yarn usage. As shown in the table, when the proportion of blue dots increases from 10% to 90%, $K_{l}$ rises markedly from 34.83% to 65.13% for Set I.

**Figure 6:**
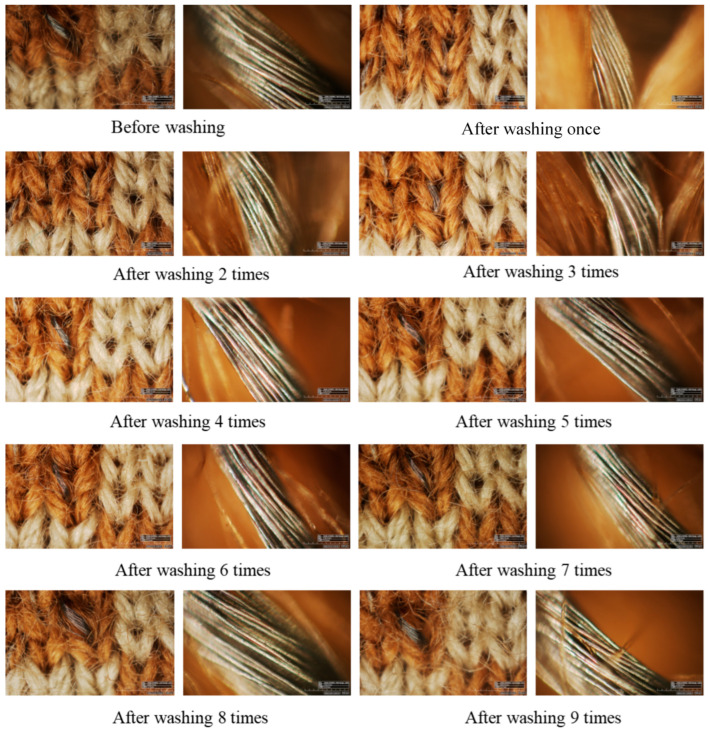
E-SEM micrographs of part of samples.

Figure 6 shows the E-SEM micrographs (RH-2000 system, HIROX, Tokyo, Japan) of the samples with 50% blue dots in Set II. Due to the magnification, only a localized region of the fabric microstructure is visible. For each wash, the left side of the sample is observed at 50× and right side at 400× magnification. The 10 different views show the same area of the same fabric to observe the change in appearance after washing. Figure 6 shows that there are no obvious changes to the surface of the conductive yarns, and the conductive yarns remain in the same configuration after being washed 10 times. It should be noted that optical microscopy mainly reveals macroscopic structural changes, while subtle surface damage or coating degradation of conductive yarns may not be fully detectable.

The resistance values for the three sets of samples were obtained through resistance testing. Table 6, Table 7 and Table 8 present the testing results for Sets I, II and III, respectively. All of the tests were conducted three times to ensure repeatable results. The values in the table are presented as mean values and SD (Standard Deviation).

A slight decrease in resistance was observed after the first washing cycle for several samples. This phenomenon may be attributed to the structural relaxation of the knitted fabric and improved contact between conductive fibers within the conductive yarns during the initial washing process. Mechanical agitation and moisture can slightly rearrange the conductive pathways, reducing contact resistance and resulting in a temporary decrease in the measured electrical resistance. It is evident that after washing, the resistance value tends to increase slightly. However, because the initial resistance values are relatively low, even a small absolute increase may correspond to a relatively large percentage change. As the consumption of the conductive yarn increases, the resistance values of each set exhibit an upward trend, which can be attributed to the longer conductive yarn length which would lead to a higher overall resistance. Moreover, even when the conductive yarn consumption is the same, fabrics with different patterns show noticeable differences in resistance. This is likely because the different patterns alter the conductive paths of the yarn, thereby affecting the amount of contact resistance between the conductive yarns, thus resulting in a significant influence of pattern variation on the resistance values [20].

By incorporating the experimental data into Equation (2), the resistance change ratio after 0–10 wash cycles for the samples can be calculated as shown in Table 9, Table 10 and Table 11. A clear trend can be observed in Table 9, Table 10 and Table 11, where the resistance of all samples gradually increases with the number of washing cycles. The increase is relatively moderate during the first five washing cycles but becomes more pronounced after approximately six cycles, indicating that cumulative mechanical abrasion during laundering progressively affects the conductive coating and the continuity of the conductive pathways. Negative resistance changes observed after the first washing cycle can be attributed to the structural relaxation of the knitted fabric and improved contact between conductive yarns caused by mechanical agitation and moisture exposure during washing. This temporary structural adjustment may reduce contact resistance at the initial stage. Comparing the three pattern sets, Set II shows the best resistance stability, whereas Set III and Set I exhibits the largest fluctuations, with a maximum resistance change of 55% after ten washing cycles. This indicates that Jacquard pattern design greatly influences the durability of electrical performance in conductive knitted fabrics.

A thermal performance evaluation was conducted following the processes in Section 2.4.3. The steady-state temperature $T_{s}$ and temperature variation $T_{v}$ are found in Table 12. It can be seen that the $T_{s}$ ranges from 35.6 to 39.67 °C, which shows a good thermal performance, with the temperatures of some of the fabrics reaching approximately 40 °C. Such a temperature range would not cause excessive thermal discomfort to the body and is considered suitable for wear comfort [38].

Although the conductive yarn content increases from 10% to 90%, the difference in steady-state temperature remains relatively small (approximately 2–3 °C). This phenomenon is related to the characteristics of the Jacquard pattern design. While the amount of conductive yarn varies greatly with different Jacquard patterns, the overall fabric size and the distribution range of the conductive area remain unchanged. The variation mainly occurs in the distribution density and spatial arrangement of conductive yarns within the same area. Therefore, the resulting change in heat generation is limited, leading to only a small variation in steady-state temperature.

## 4. Evaluation of Wearability and Electrical Performance

### 4.1. Definition of Evaluation Indicators

Table 13 provides the definition of $x_{1}$, $x_{2}$ and $x_{3}$ for the evaluation system. In the evaluation system, $x_{1}$ is treated as a negative indicator because increased conductive yarn consumption leads to higher material cost, whereas lower yarn usage is more favorable from the perspective of economic efficiency and resource utilization.

The indicators $x_{1}$, $x_{2}$ and $x_{3}$ are physically related to the structural design of the Jacquard pattern, particularly the proportion of conductive yarn used in the fabric. Indicator $x_{1}$ represents the consumption of conductive yarn, which is directly associated with the color proportion in the Jacquard pattern; a larger pattern proportion corresponds to a higher amount of conductive yarn used in the fabric. Indicator $x_{2}$ represents the electrical resistance of the conductive fabric. As the proportion of conductive yarn in the pattern increases, the conductive pathway within the fabric becomes longer, leading to an increase in electrical resistance. Indicator $x_{3}$ represents the steady-state temperature of the fabric during the heating process, which reflects the overall thermal performance of the conductive knitted structure.

Although these indicators are structurally related through the Jacquard pattern design, they characterize different aspects of the conductive textile system, namely material consumption, electrical performance, and thermal response. Therefore, they provide complementary information for the fuzzy comprehensive evaluation.

### 4.2. Standards for Evaluating Grade

Five grades for evaluation were established based on experimental data distribution and application requirements, as shown in Table 14. The classification criteria presented in Table 14 were established based on the distribution of the experimental data. For each evaluation indicator, the observed data range obtained from the experiments was divided into five levels (Excellent, Good, Medium, Pass, and Fail) using an equal-interval classification method. In terms of performance interpretation, a lower conductive yarn consumption ratio corresponds to reduced material cost, while a smaller resistance change rate after washing indicates better electrical stability and wash durability of the conductive knitted fabrics. Therefore, the “Excellent” level represents samples that achieve a balanced optimization of low material consumption, good thermal insulation performance, and minimal resistance variation after repeated washing.

By adopting the methodology in Section Determination of Indicator Weights with Entropy Weighing, the indicators $e_{j}$, $g_{j}$ and $W_{j}$ can be calculated as shown in Table 15. It can be seen in the table that the total difference coefficient is the sum of different coefficient $g_{j}$, and its calculated value is 1.68.

### 4.3. Calculation of Degree of Membership

The membership matrix B is obtained by combining the weight W with the fuzzy matrix R, which can be expressed as:

(13) $$B=W\cdot R=\left[0.3444,0.3285,0.3271\right]\;[\begin{array}{ccccc} u_{11} & u_{12} & u_{13} & u_{14} & u_{15}\\ u_{21} & u_{22} & u_{23} & u_{24} & u_{25}\\ u_{31} & u_{32} & u_{33} & u_{34} & u_{35} \end{array}]$$

Each element of B represents the weighted sum of the degree of membership of the three indicators under a specific evaluation grade, which is a 1 × 5 vector.

### 4.4. Determination of Final Evaluation Grade

According to the maximum membership principle, the evaluation grade that corresponds to the largest value in the comprehensive membership vector B is taken as the final grade of the sample. The sum of the elements in B equals 1, reflecting the normalized membership distribution.

If two grades have equal membership values, the final decision is made by considering indicator weight and actual performance, with priority given to the grade associated with the indicator of a higher weight. For example, if (B=[0.8,0.1,0.05,0.03,0.02]), the sample is classified as “Excellent”. When the membership values of two grades are equal (e.g., B=[0.4,0.4,0.1,0.05,0.05]), the following decision rule is applied: first, identify which indicator contributes the most to the calculation of each of the two grades; then compare the weights of these indicators. The grade corresponding to the indicator with the larger weight is taken as the final evaluation result [39].

**Figure 7:**
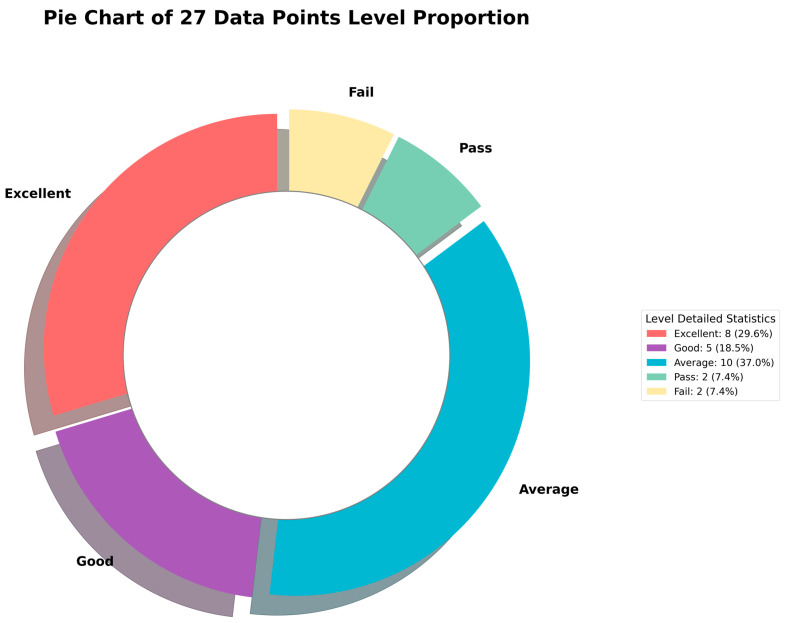
Pie chart of data point level distribution of 27 samples.

The distribution of the 27 samples, as illustrated by the pie chart in Figure 7, indicates that approximately 30% of the samples have an excellent performance, characterized by a high conductive yarn content, stable electrical resistance, and a favorable temperature response. Samples classified as having good performance slightly outnumber those with excellent performance, and together, they account for 48.1% of the total samples. While a lower content of conductive yarn is indeed associated with lower costs, a slightly higher content was found to provide the best overall performance, including better electrical resistance stability and a higher temperature rise. The results indicate that a substantial proportion of the samples demonstrated good or excellent performance, while most samples still met the basic functional requirements.

Most of the samples have a average performance, thus indicating that they meet the basic functional requirements but have not yet reached the optimized state. The proportion of samples with a passing performance is comparable to that of the samples with an excellent performance, thus implying that only a subset of the samples meets the minimum performance criteria and so there is still considerable potential for further improvement. Samples that have a failing performance constitute a relatively small percentage (7.4%), and typically associated with an extremely low conductive yarn consumption ratio ($x_{1}$) and/or excessively high rates of change in resistance ($x_{2}$), which result in abnormal temperature increase or electrical failure.

Overall, the samples with an excellent performance characterize a bit more conductive yarn, show little variation in resistance, and have a stable temperature, so that these constitute as the elements for optimizing the production process.

**Figure 8:**
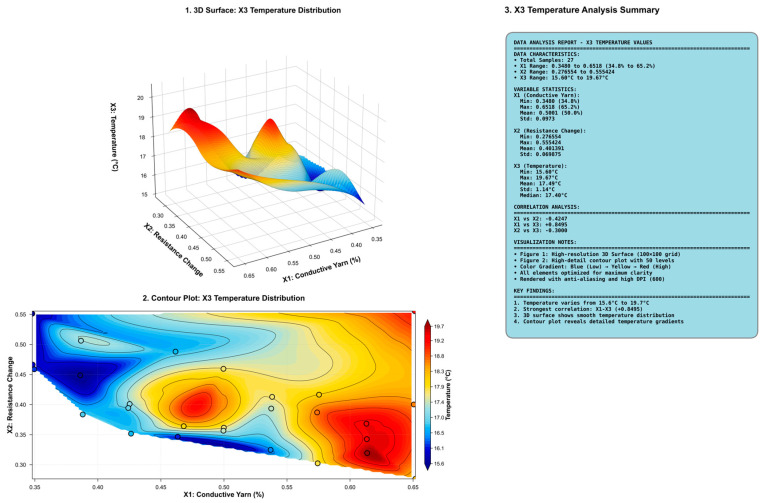
3D surface and contour maps that show temperature distribution.

Figure 8 shows the 3D surface and contour maps that show the temperature distribution. The figure illustrates the combined effects of conductive yarn consumption ratio ($x_{1}$) and rate of change in resistance ($x_{2}$) on the temperature ($x_{3}$). The contour plot of the temperature distribution on the $x_{1}$–$x_{2}$ plane shows a trend of temperature increase with higher $x_{1}$ values, with the contour lines extending approximately in a diagonal direction. That is, there is a pronounced upward trend along the $x_{1}$ axis, which indicates that the temperature show an increasing trend with increasing conductive yarn content, which confirms a positive relationship between $x_{1}$ and $x_{3}$. In contrast, variations along the $x_{2}$ direction are relatively moderate, thus suggesting that the influence of the rate of change in resistance on temperature is comparatively weaker. That is, the relatively sparse contour spacing along the $x_{2}$ axis indicates that temperature is less sensitive to variations in the rate of change in resistance, thus further corroborating an important role of amount of conductive yarn in determining the thermal behavior of conductive knitted fabrics.

**Figure 9:**
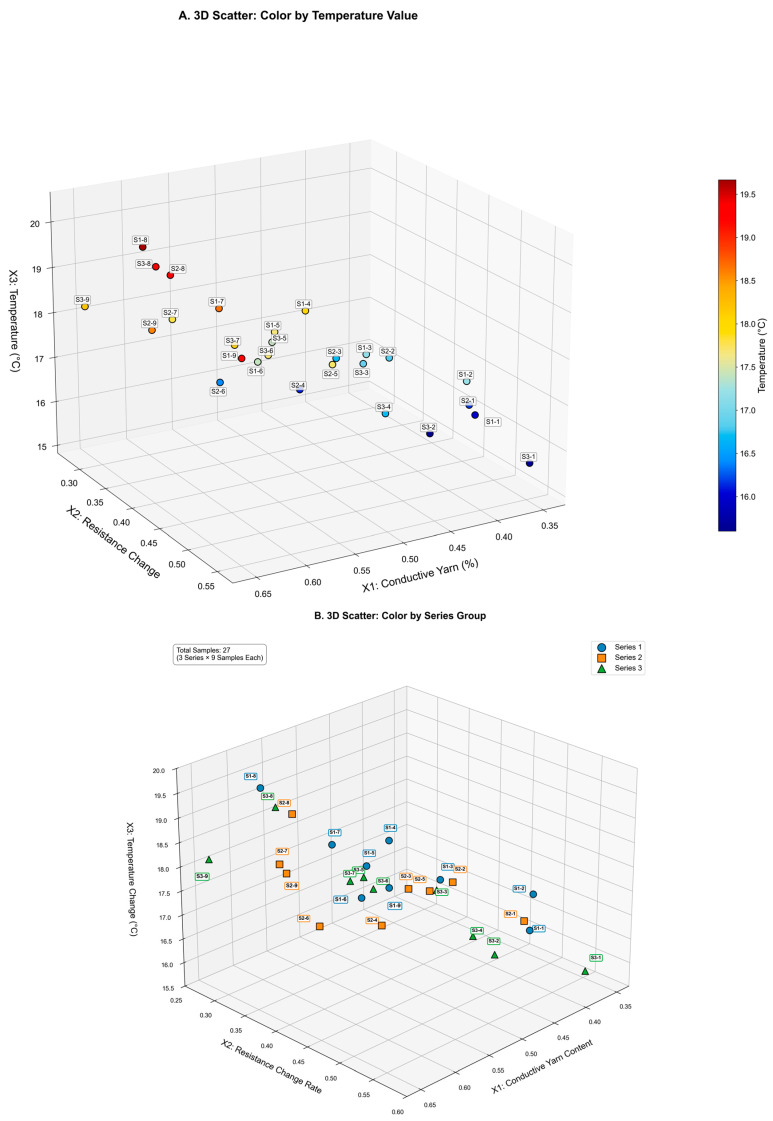
3D scatter plot of 27 samples distributed across the $x_{1}$–$x_{2}$–$x_{3}$ feature space colored by: (**A**) temperature value and (**B**) sets of samples.

The 3D scatter plot further presents the actual distribution of the 27 samples in the $x_{1}$–$x_{2}$–$x_{3}$ space (Figure 9). High-temperature samples are mainly found in regions with high $x_{1}$ and low $x_{2}$, which is consistent with the pattern of the surface map. This observation further confirms that $x_{1}$ is the dominant factor that governs temperature response, whereas $x_{2}$ is secondary.

Among the three sets of samples, Set I exhibits the best overall performance, with the highest proportion of samples that have excellent and good performances (66.7%: 6 out of 9 samples), with a relatively high percentage of excellent-grade samples. The sample distribution across the performance levels is well balanced, with all grades represented, thus indicating good process robustness and stability.

Set II shows a stable and well-controlled performance, with all samples meeting the qualification criteria and no unqualified samples observed, thus suggesting the most stringent process control among the three sets of samples. However, the percentage of samples with excellent–good performance (33.3%: 3 out of 9 samples) is lower than that of Set I, thus indicating that while this process readily meets baseline requirements, achieving an exceptional performance remains challenging.

In contrast, Set III is at a medium level. The percentage of samples with excellent–good performance is 44.4% (4 out of 9 samples). Although this proportion was higher than that of the second group, it was still lower than that of the top-performing first group. Moreover, there were some samples that were classified as unqualified, indicating that there is still room for improvement in the stability of the process parameter control, and performance fluctuations are prone to occur.

The experimental results indicate that samples with slightly higher conductive yarn content exhibit more stable electrical resistance and a higher temperature rise. Based on the distribution characteristics of the evaluated samples, it is recommended that $x_{1}$ be controlled within the range of 38–54%, where there are the most samples and process stability is maximized, thus providing a favorable balance between performance reliability and manufacturing robustness. It represents a balanced range that ensures relatively low cost while achieving stable electrical and thermal performance.

### 4.5. Discussion and Limitation

To further evaluate whether significant differences exist among the three sample sets, a Kruskal–Wallis non-parametric test was conducted. This statistical method is suitable for comparing multiple independent groups when the sample size is relatively small or when the assumption of normal distribution may not be satisfied. The results show that the differences among the three groups are not statistically significant (H = 0.814, *p* = 0.666 > 0.05). Although the descriptive statistics suggest that Set I exhibits slightly higher performance levels than Set II and III, the statistical analysis indicates that these variations do not reach statistical significance. Therefore, the observed differences among the three groups may be attributed to random variation rather than inherent group effects.

Nevertheless, the experimental results still reveal certain trends in the electrothermal performance of the jacquard conductive knitted fabrics. In particular, the washing durability and heating performance appear to be closely related to the proportion of conductive yarn in the fabric structure. Fabrics with an appropriate conductive yarn content generally exhibit more stable electrical resistance after washing and a more pronounced temperature rise during heating tests. This indicates that conductive yarn content plays a crucial role in determining both the electrical conductivity and the stability of the heating response during repeated use. The fuzzy comprehensive evaluation integrates multiple indicators, including the conductive yarn usage ratio, resistance variation after washing, and temperature variation, providing a systematic assessment of the overall performance. The results demonstrate that samples with a moderate conductive yarn content tend to achieve better overall performance, indicating that balancing material composition and heating properties is essential for the design of practical conductive knitted heating textiles.

Despite the promising results obtained in this study, several limitations should be acknowledged. First, the number of samples investigated was relatively limited, which may restrict the statistical representativeness of the results. Second, the washing and heating performance evaluation was mainly based on resistance variation after washing and temperature rise under controlled laboratory conditions, while long-term durability and real wearing conditions were not investigated. In addition, the fuzzy comprehensive evaluation method relies on the selection of evaluation indicators and their corresponding weights, which may introduce a certain degree of subjectivity. Future studies could expand the sample size, incorporate additional performance indicators such as comfort and mechanical durability, and conduct long-term wear tests to further validate the applicability of the proposed conductive knitted fabrics.

## 5. Conclusions

In this work, the samples are systematically investigated from the perspective of engineering applicability for wearable electronics, with a particular focus on washing durability, and electrical stability under practical use conditions. To address the limitations of conventional evaluation approaches, a comprehensive fuzzy logic method is introduced to integrate a number of performance indicators into a unified and quantitative assessment framework. The conclusions are summarized as follows:

(1) With the same knitting parameters, the fabric density of the samples remains within a narrow range, and the conductive yarns have the same configuration after being washed 10 times, with no obvious changes to the surface of the conductive yarns.
(2) Different sets of Jacquard pattern samples with the same amount of conductive yarn confirm that the pattern design and structural configuration play a critical role in determining electrical stability and durability, even when the conductive yarn consumption remains the same.
(3) A statistical distribution of the performance reveals that 48.1% of the samples have an excellent or good performance, while only 7.4% fail, thus indicating a generally high level of process reliability. As the proportion of conductive yarn increases, both the resistance and temperature show an upward trend. This indicates that the conductive yarn content is the dominant factor governing temperature response behavior, whereas the rate of change in resistance plays a secondary role.

Overall, this study shows that the comprehensive fuzzy logic method is an effective engineering-oriented approach for performance assessment and decision-making with conductive knitted fabrics, particularly for structurally complex Jacquard designs. The proposed methodology offers practical guidance for pattern design, structure selection and material utilization in wearable electronics, thus contributing to the development of durable, reliable, and scalable conductive textile systems. These fabrics show potential for use in wearable heating garments and other smart textile applications where stable electrothermal performance and washing durability are required. Future work should focus on long-term cyclic loading, real user conditions, and system-level integration to further enhance the applicability of conductive Jacquard knitted fabrics in smart wearable products.

## Figures and Tables

**Table 1:** Loop length of conductive yarn ($L_{c}$) and normal yarn ($L_{n}$) within the fabric samples.

		10%	20%	30%	40%	50%	60%	70%	80%	90%
Set I	$L_{c}$	29.93	34.51	39.22	44.56	48.52	52.33	56.46	60.93	65.35
	$L_{n}$	65.31	61.02	56.78	52.13	48.52	43.48	39.09	34.37	29.96
Set II	$L_{c}$	30.23	34.90	39.46	43.87	48.1	52.29	56.91	61.11	65.56
	$L_{n}$	65.59	61.43	57.20	52.75	48.07	43.45	39.18	34.56	30.18
Set III	$L_{c}$	29.88	34.42	38.77	43.17	48.00	52.22	56.66	61.02	65.31
	$L_{n}$	65.24	60.91	56.56	52.08	48.03	43.31	38.98	34.50	29.94

**Table 2:** Specifications of yarn materials for experiments.

Material	Yarn Count/Tex	Composition	Linear Resistance/Ω per cm	Supplier
Ordinary Yarn A/B	41.67	100% merino wool	/	Xinao Textiles Co., Ltd., Jiaxing, China
Conductive Yarn A	47	Nylon 66 coated with silver	1.07	Statex Produktions-und Verriebs GmbH, Bremen, Germany
Conductive Yarn B	7.78	Nylon 66 coated with silver	6.80	Qingdao Hengtong X-Silver Speciality Textile Co., Ltd., Qingdao, China

**Table 3:** Machine settings for knitting samples.

Parameter	Setting	Comments
Gauge	12	Shima Seiki computerized flat-knitting machine
Yarn Tension	2 (machine value)	Kept constant for all samples
Take-down Rate	50 (machine value)	Standard setting to maintain fabric stability
Knitting Speed	0.5 m/s	Uniform speed to minimize structural differences
Stitch Cam Setting	45 (machine value)	Ensures consistent loop size

**Table 4:** Course and wale densities of samples (Unit: loops per cm).

	Density	10%	20%	30%	40%	50%	60%	70%	80%	90%
Set I	Course	8.1	7.9	8.1	8.0	8.1	8.0	8.1	8.0	8.1
	Wale	11.2	11.3	10.8	11.1	11.0	11.2	11.1	11.3	11.0
Set II	Course	7.8	7.9	8.1	8.0	7.9	8.1	8.0	8.1	7.9
	Wale	10.6	10.8	10.9	11.0	10.9	11.1	11.0	10.8	11.1
Set III	Course	8.3	8.2	8.1	8.2	8.0	8.1	8.2	8.0	8.1
	Wale	11.4	11.3	11.2	11.1	11.2	11.0	11.3	11.1	11.2

**Table 5:** Conductive yarn consumption ratio for three sets of samples.

	10%	20%	30%	40%	50%	60%	70%	80%	90%
Set I	34.83	38.66	42.52	46.81	49.99	53.75	57.39	61.36	65.13
Set II	34.96	38.80	42.63	46.33	49.96	53.70	57.43	61.30	65.05
Set III	34.80	38.60	42.41	46.16	49.96	53.82	57.54	61.33	65.18

**Table 6:** Resistance values after washing: Set I (Unit: Ω).

No. of Washes	10%		20%		30%		40%		50%		60%		70%		80%		90%	
	Mean	SD	Mean	SD	Mean	SD	Mean	SD	Mean	SD	Mean	SD	Mean	SD	Mean	SD	Mean	SD
0	1.0136	0.0011	1.0274	0.0017	1.1796	0.0041	1.3872	0.0013	1.4983	0.0027	1.5561	0.0029	1.6569	0.0022	1.7252	0.0023	2.6535	0.0058
1	1.0381	0.0010	1.0196	0.0023	1.1489	0.0029	1.41867	0.0016	1.5331	0.0012	1.5863	0.0006	1.6803	0.0056	1.7083	0.0053	2.8073	0.0055
2	1.0514	0.0023	1.0534	0.0006	1.2290	0.0058	1.43867	0.0018	1.5778	0.0025	1.6571	0.0087	1.7358	0.0036	1.7633	0.0042	3.0143	0.0055
3	1.0764	0.0030	1.0586	0.0006	1.2649	0.0027	1.43837	0.0007	1.5733	0.0024	1.6537	0.0044	1.7819	0.0057	1.8922	0.0069	3.1432	0.0047
4	1.1760	0.0051	1.1488	0.0025	1.3247	0.0062	1.54167	0.0055	1.6648	0.0070	1.7380	0.0057	1.8537	0.0073	1.9184	0.0078	3.3090	0.0079
5	1.1685	0.0041	1.1624	0.0052	1.3661	0.0045	1.56433	0.0055	1.7013	0.0055	1.7658	0.0080	1.8974	0.0045	1.9538	0.0043	3.4329	0.0063
6	1.4612	0.0027	1.4028	0.0045	1.6176	0.0046	1.79077	0.0049	1.9435	0.0039	2.0184	0.0048	2.1650	0.0071	2.2115	0.0057	3.6560	0.0098
7	1.4100	0.0041	1.3787	0.0096	1.5716	0.0094	1.76907	0.0067	1.9184	0.0070	2.0187	0.0071	2.1266	0.0052	2.1760	0.0092	3.6513	0.0105
8	1.4263	0.0058	1.4785	0.0019	1.5977	0.0040	1.78013	0.0055	1.9847	0.0122	2.0562	0.0092	2.1857	0.0117	2.1885	0.0097	3.7508	0.0106
9	1.3748	0.0078	1.4259	0.0102	1.5407	0.0088	1.77907	0.0096	1.9075	0.0087	2.0213	0.0104	2.1242	0.0075	2.1407	0.0097	3.9199	0.0160
10	1.4864	0.0053	1.5475	0.0038	1.6526	0.0049	1.89223	0.0062	2.0398	0.0025	2.1683	0.0015	2.2982	0.0038	2.2761	0.0051	4.1272	0.0065

**Table 7:** Resistance values after washing for SetII (Unit: Ω).

No. of Washes	10%		20%		30%		40%		50%		60%		70%		80%		90%	
	Mean	SD	Mean	SD	Mean	SD	Mean	SD	Mean	SD	Mean	SD	Mean	SD	Mean	SD	Mean	SD
0	1.0629	0.0008	1.1212	0.0018	1.2370	0.0018	1.34293	0.0005	1.4719	0.0058	1.5779	0.0025	1.6901	0.0016	1.8734	0.0039	1.9742	0.0042
1	0.9795	0.0009	1.0501	0.0009	1.2340	0.0022	1.32830	0.0045	1.4443	0.0044	1.5212	0.0003	1.6329	0.0006	1.8731	0.0036	1.9424	0.0039
2	1.0111	0.0017	1.1040	0.0017	1.2570	0.0017	1.37657	0.0020	1.4758	0.0050	1.5809	0.0064	1.7329	0.0045	1.9505	0.0032	2.0290	0.0032
3	1.0108	0.0003	1.1138	0.0027	1.2774	0.0070	1.40007	0.0036	1.5355	0.0032	1.6360	0.0007	1.7575	0.0085	1.9761	0.0056	2.0209	0.0038
4	1.1082	0.0040	1.1980	0.0011	1.3703	0.0066	1.47610	0.0037	1.6397	0.0064	1.7563	0.0033	1.8067	0.0002	2.0241	0.0056	2.1391	0.0050
5	1.1123	0.0040	1.2047	0.0059	1.4002	0.0042	1.53247	0.0060	1.6489	0.0046	1.8049	0.0061	1.8577	0.0058	2.0783	0.0077	2.2843	0.0035
6	1.3723	0.0029	1.4583	0.0004	1.6479	0.0054	1.79077	0.0049	1.9320	0.0126	2.0337	0.0027	2.1183	0.0047	2.3380	0.0091	2.5684	0.0128
7	1.3425	0.0067	1.4130	0.0064	1.6082	0.0050	1.76417	0.0080	1.9222	0.0086	1.9947	0.0078	2.0671	0.0052	2.3300	0.0092	2.4466	0.0082
8	1.3968	0.0037	1.4147	0.0042	1.6171	0.0049	1.75880	0.0083	1.9824	0.0113	1.9934	0.0044	2.1078	0.0067	2.3758	0.0069	2.4822	0.0087
9	1.3350	0.0052	1.3763	0.0059	1.5962	0.0050	1.70580	0.0072	1.9930	0.0068	1.9474	0.0096	2.0515	0.0079	2.4146	0.0077	2.5181	0.0120
10	1.4350	0.0043	1.4639	0.0042	1.6726	0.0013	1.79227	0.0009	2.0608	0.0060	2.0553	0.0054	2.1761	0.0050	2.5300	0.0022	2.6915	0.0053

**Table 8:** The resistance values after washing for Sets III (Unit: Ω).

No. of Washes	10%		20%		30%		40%		50%		60%		70%		80%		90%	
	Mean	SD	Mean	SD	Mean	SD	Mean	SD	Mean	SD	Mean	SD	Mean	SD	Mean	SD	Mean	SD
0	1.0747	0.0057	1.2012	0.0056	1.2897	0.0025	1.42383	0.0017	1.5467	0.0116	1.6955	0.0013	1.8389	0.0017	1.9438	0.0035	2.0536	0.0080
1	1.0291	0.0067	1.1436	0.0034	1.2807	0.0048	1.38490	0.0034	1.5062	0.0122	1.7070	0.0012	1.8206	0.0006	1.9174	0.0011	2.0467	0.0031
2	1.0483	0.0017	1.1882	0.0014	1.2791	0.0034	1.46783	0.0021	1.5567	0.0047	1.7522	0.0052	1.9337	0.0036	2.0362	0.0045	2.0967	0.0040
3	1.1339	0.0016	1.2312	0.0017	1.3030	0.0029	1.48417	0.0037	1.5781	0.0005	1.7886	0.0044	1.9463	0.0051	2.0926	0.0105	2.1096	0.0034
4	1.2310	0.0060	1.3404	0.0025	1.3825	0.0046	1.59220	0.0065	1.6554	0.0072	1.9851	0.0080	2.0829	0.0027	2.1553	0.0029	2.1930	0.0042
5	1.2223	0.0017	1.3662	0.0022	1.3909	0.0046	1.62260	0.0067	1.6823	0.0045	2.0049	0.0032	2.1130	0.0072	2.1911	0.0058	2.2532	0.0058
6	1.5001	0.0017	1.6163	0.0057	1.6444	0.0031	1.86567	0.0056	1.9281	0.0025	2.2642	0.0071	2.3632	0.0061	2.4483	0.0032	2.5119	0.0075
7	1.4683	0.0030	1.5790	0.0053	1.6241	0.0066	1.86297	0.0086	1.9051	0.0059	2.1962	0.0073	2.3729	0.0057	2.4247	0.0077	2.4836	0.0104
8	1.5984	0.0018	1.5671	0.0037	1.6285	0.0031	1.94617	0.0080	1.9255	0.0040	2.2287	0.0044	2.4536	0.0079	2.4316	0.0061	2.4621	0.0141
9	1.4863	0.0037	1.5673	0.0115	1.5718	0.0018	1.95533	0.0094	1.8765	0.0100	2.2077	0.0138	2.4157	0.0116	2.4274	0.0118	2.4335	0.0122
10	1.5757	0.0012	1.6525	0.0018	1.7494	0.0013	2.05833	0.0013	2.0028	0.0030	2.3594	0.0060	2.5789	0.0071	2.5894	0.0079	2.6195	0.0094

**Table 9:** Washing resistance ratio after 0–10 wash cycles: Set I.

No. of Washes	10%	20%	30%	40%	50%	60%	70%	80%	90%
1	2.4163	−0.7631	−2.6009	2.2686	2.3584	1.9388	1.4092	−0.9808	5.7963
2	3.7324	2.5277	4.1870	3.7103	5.2977	6.4873	4.7587	2.1899	13.5888
3	6.2030	3.0309	7.2288	3.6894	5.0046	6.2714	7.5428	9.6726	18.4584
4	16.0278	11.8161	12.3009	11.1317	11.0335	11.6868	11.8706	11.2006	24.7069
5	15.2908	13.1359	15.8106	12.7674	13.5114	13.4726	14.5104	13.2485	29.3748
6	44.1685	36.5326	37.1290	29.0908	29.6985	29.7111	30.6587	28.1895	37.7813
7	39.1091	34.1908	33.2260	27.5250	27.9797	29.7233	28.3430	26.1306	37.6049
8	40.7252	43.9094	35.4403	28.3245	32.2972	32.1357	31.9098	26.8534	41.3574
9	35.6361	38.7820	30.6166	28.2459	27.1811	29.8897	28.1976	24.0856	47.7299
10	46.6479	50.6205	40.1046	36.4033	36.1588	39.3440	38.7000	31.9352	55.5424

**Table 10:** Washing resistance ratio after 0–10 wash cycles: Set II.

No. of Washes	10%	20%	30%	40%	50%	60%	70%	80%	90%
1	−0.1019	−0.4735	−0.3234	−0.3759	2.2727	−1.7431	−2.0996	1.2439	1.0943
2	3.0581	4.4508	1.6977	3.3835	4.4034	1.7431	3.6593	5.4083	5.5758
3	3.0581	5.3030	2.8294	4.9624	8.8068	5.6165	5.0390	6.5982	5.2110
4	12.6402	13.4470	10.3476	10.8271	16.0511	13.2343	8.3383	9.2482	11.3080
5	13.0479	13.5417	12.9345	14.8120	16.6903	16.2686	11.1578	12.1147	18.8640
6	39.5515	38.1629	32.8213	34.3609	36.2216	31.1814	26.7546	26.0681	33.2465
7	36.2895	33.3333	29.6686	32.1805	35.8665	28.3409	23.8752	25.5814	27.1496
8	42.0999	33.6174	30.3961	31.6541	39.9148	28.4700	26.0948	28.1774	28.9734
9	35.7798	29.9242	28.6985	27.7444	40.9801	25.1775	22.6155	30.2866	30.6410
10	45.8716	38.3523	35.1657	34.6617	45.9517	32.4726	30.2340	36.8307	40.0208

**Table 11:** Washing resistance ratio after 0–10 wash cycles: Set III.

No. of Washes	10%	20%	30%	40%	50%	60%	70%	80%	90%
1	0.9843	0.4382	1.8327	0.0723	1.4237	2.2768	−0.8242	−0.5189	0.0979
2	3.2480	4.0316	1.8327	6.0738	5.2881	4.8532	6.0989	5.5008	2.5453
3	11.3189	7.7125	3.6653	7.2307	6.9831	7.0701	6.6484	8.3031	3.1816
4	20.9646	17.3532	9.9602	15.0398	11.8644	18.6339	14.4505	11.7800	7.2442
5	20.1772	19.6319	10.7570	17.0644	13.8305	20.0120	15.8791	13.5444	10.1322
6	47.6378	41.4549	30.9163	34.7072	30.6441	35.4703	29.6703	26.9850	22.7117
7	44.4882	38.1245	29.1633	34.4902	28.8136	31.3960	30.2747	25.5838	21.3412
8	57.2835	37.2480	29.6414	40.1302	30.3729	33.4332	34.6154	26.0509	20.2154
9	46.2598	36.9851	25.1793	40.9255	26.7119	31.8754	32.4176	25.6876	18.7959
10	55.1181	44.8729	39.4422	48.8069	35.6610	41.2822	41.6484	34.2501	27.6554

**Table 12:** Steady-state temperature and temperature variation after 10 washes. (Unit: °C).

Conductive Yarn Content (%)	$\mathrm{Set}\;I\;\mathrm{Surface}\;\mathrm{Temperature}\;T_{s}$	$\mathrm{Temperature}\;\mathrm{Rise}\;T_{v}$	$\mathrm{Set}\;\mathrm{II}\;\mathrm{Surface}\;\mathrm{Temperature}\;T_{s}$	$\mathrm{Temperature}\;\mathrm{Rise}\;T_{v}$	$\mathrm{Set}\;\mathrm{III}\;\mathrm{Surface}\;\mathrm{Temperature}\;T_{s}$	$\mathrm{Temperature}\;\mathrm{Rise}\;T_{v}$
10%	36	16	36.2	16.2	35.6	15.6
20%	37.2	17.2	36.8	16.8	35.6	15.6
30%	37.2	17.2	36.7	16.7	36.9	16.9
40%	38	18	36.1	16.1	36.7	16.7
50%	37.7	17.7	37.7	17.7	37.4	17.4
60%	37.4	17.4	36.4	16.4	37.7	17.7
70%	38.7	18.7	37.8	17.8	38.1	18.1
80%	39.7	19.7	39.4	19.4	39.4	19.4
90%	39.2	19.2	38.6	18.6	38.2	18.2

Note: $T_{s}$ represents the steady-state surface temperature under electrical heating. The temperature rise $T_{v}$ is defined as $T_{v}$ = $T_{s}$ − $T_{0}$, where the initial ambient temperature $T_{0}$ is 20 °C.

**Table 13:** Definition of evaluation indicators.

Indicator	Designation	Attribute	Experimental Range	Result	Ideal Value
Conductive yarn consumption ratio ($K_{l}$)	$x_{1}$	Negative	34.80~65.18%	Cost of conductive yarns	<38%
Rate of change in resistance after 10 washes ($R_{w})$	$x_{2}$	Negative	27.66~55.54%	Washing durability and stability	≤32%
Temperature variation (${\;T}_{v})$	$x_{3}$	Positive	15.6–19.67 °C	Thermal performance of fabrics	≥17.4 °C

**Table 14:** Standards for evaluating grade: $x_{1}$, *x*_2_ and *x*_3_.

Grade	*x*_1_ (%)	*x*_2_ (%)	*x*_3_ (°C)
Excellent	<38	≤32	≥17.4
Good	38–46	32–38	16.8–17.4
Average	46–54	38–44	16.2–16.8
Pass	54–62	44–50	15.6–16.2
Fail	>62	>50	<15.6

**Table 15:** $e_{j}$, $g_{j}$ and $W_{j}$ values for $x_{1}$, $x_{2}$ and $x_{3}$.

Indicator	$e_{j}$	$g_{j}$	$W_{j}$
$x_{1}$	0.4205	0.5795	0.3444
$x_{2}$	0.4472	0.5528	0.3285
$x_{3}$	0.4497	0.5503	0.3271

## Data Availability

The original contributions presented in this study are included in the article. Further inquiries can be directed to the corresponding author.
